# Multiple marker abundance profiling: combining selected reaction monitoring and data‐dependent acquisition for rapid estimation of organelle abundance in subcellular samples

**DOI:** 10.1111/tpj.13743

**Published:** 2017-11-20

**Authors:** Cornelia M. Hooper, Tim J. Stevens, Anna Saukkonen, Ian R. Castleden, Pragya Singh, Gregory W. Mann, Bertrand Fabre, Jun Ito, Michael J Deery, Kathryn S. Lilley, Christopher J. Petzold, A. Harvey Millar, Joshua L. Heazlewood, Harriet T. Parsons

**Affiliations:** ^1^ ARC Centre of Excellence in Plant Energy Biology The University of Western Australia Perth WA 6009 Australia; ^2^ MRC Laboratory of Molecular Biology Cambridge CB2 0QH UK; ^3^ Department of Biochemistry University of Cambridge Cambridge CB2 1QR UK; ^4^ Joint BioEnergy Institute Lawrence Berkeley National Laboratory Berkeley CA 94702 USA; ^5^ School of BioSciences The University of Melbourne Melbourne VIC 3010 Australia; ^6^ Copenhagen University, Plant and Environmental Sciences Frederiksberg 1871 Denmark

**Keywords:** Arabidopsis, organelles, tissues, protein abundance, shotgun proteomics, selected reaction monitoring

## Abstract

Measuring changes in protein or organelle abundance in the cell is an essential, but challenging aspect of cell biology. Frequently‐used methods for determining organelle abundance typically rely on detection of a very few marker proteins, so are unsatisfactory. *In silico* estimates of protein abundances from publicly available protein spectra can provide useful standard abundance values but contain only data from tissue proteomes, and are not coupled to organelle localization data. A new protein abundance score, the normalized protein abundance scale (NPAS), expands on the number of scored proteins and the scoring accuracy of lower‐abundance proteins in Arabidopsis. NPAS was combined with subcellular protein localization data, facilitating quantitative estimations of organelle abundance during routine experimental procedures. A suite of targeted proteomics markers for subcellular compartment markers was developed, enabling independent verification of *in silico* estimates for relative organelle abundance. Estimation of relative organelle abundance was found to be reproducible and consistent over a range of tissues and growth conditions. *In silico* abundance estimations and localization data have been combined into an online tool, multiple marker abundance profiling, available in the SUBA4 toolbox (http://suba.live).

## Significance Statement

Accurately determining the subcellular composition of complex samples is difficult, especially as organelle proteomes change in response to their environment. Here we improve the speed and accuracy of subcellular analyses by developing methods which bridge the gap between the analytical benefits of mass spectrometry and its wider availability within the Arabidopsis research community, and present a template for development in other species.

## Introduction

Understanding how protein abundance relates to protein characteristics such as location, function or post‐translational modification is an important aspect of understanding biological systems, but reliably estimating protein abundance is non‐trivial. Assessing expression of protein‐coding genes is facilitated by microarray data, or directly measured by quantitative polymerase chain reaction and RNA sequencing. However, this informs little about actual protein abundance, as global protein expression studies show inconsistent correlation with gene expression (Greenbaum *et al*., 2003; Gry *et al*., 2009). The development of mass spectrometry‐based protein profiling, or proteomics, has provided an analytical platform that enables the estimation of protein abundance from a biological sample. Relative quantitation of *in vivo* protein abundance is now possible using quantitative mass spectrometry of labelled proteins (Thompson *et al*., 2003; Ross *et al*., 2004; Christoforou *et al*., 2016). Although accurate, such approaches are expensive. Label‐free proteomics (Cox and Mann, 2008; Arike and Peil, 2014) offers a cheaper, only moderately less accurate option, but still requires access to specialized equipment, software and expertise. Difficulties in obtaining comparative protein abundance data can be bypassed by referring to standard abundance values derived from publicly available mass spectrometry data, such as has been done for Arabidopsis at paxdb.org (Wang *et al*., 2012a). However, *in silico* values cannot describe changes in protein or organelle abundance in response to external factors. Furthermore, low‐abundance proteins that are poorly represented in whole‐tissue proteomes often have large errors associated with abundance values, or are missing altogether. Monitoring low‐abundance proteins is particularly important as these can be critical in localized responses to environmental perturbations. Although quantitative mass spectrometry techniques can deliver appreciable coverage of low‐abundance organelles (Thelen and Peck, 2007; Nikolovski *et al*., 2012; Groen *et al*., 2014), reducing sample complexity through organelle enrichment remains the approach of choice for identifying very‐low‐abundance proteins. A fundamental consideration when conducting organelle enrichments for proteomic surveys is how best to estimate contamination from other cellular compartments. Enzyme activity assays and immunoblotting on purified organelles and whole‐tissue extracts are typical measures of organelle purity levels, but results are hard to quantify as, even with careful sample handling, variation and bias can easily be introduced (Taylor and Posch, 2014). Furthermore, the limited number of commercially available antibodies against proteins from plant species means that conclusions must be drawn from one or a few proteins in most cases. For studies aiming at very‐high‐purity organelle preparations, antibodies give inadequate information. This offers a poor overview for researchers wanting to assess the subcellular composition of tissue homogenates (Fernandez‐Calvino *et al*., 2011; Parsons *et al*., 2012), or assess the effect of environmental stimuli (Teng *et al*., 2006; Keech *et al*., 2007; Lee *et al*., 2008) or mutations (Orth *et al*., 2007; Pan *et al*., 2016) on organelle abundance and composition. Targeted proteomic approaches such as selected reaction monitoring (SRM) have provided a valuable alternative for monitoring proteins of interest (Lehmann *et al*., 2008; Taylor *et al*., 2014). SRM has also been used to assess organelle contamination in cytosolic‐enriched fractions of Arabidopsis (Ito *et al*., 2011), and its broad applicability in estimating organelle profiles from plant extracts has been outlined (Parsons and Heazlewood, 2015). However, for some research groups organelle enrichments are performed as part of standard methodologies for a wide range of biological questions. Where protein abundance is not the primary question, approaches such as SRM may be technically prohibitive or excessive, and access to specialized mass spectrometers may be limiting. Rather, it would suffice to use improved *in silico* estimation of standard protein abundance values, combined with rapid screening for organelle purity from shotgun analyses of enriched fractions. Shotgun analysis is a standard, straightforward, relatively high‐throughput technique that can be easily outsourced if in‐house facilities are lacking, and is often obtained already for other experimental reasons. If *in silico* protein abundance estimates could be combined with localization information, this could be used to produce an instant, quantitative estimate of relative organelle abundance from a single shotgun mass spectrometry experiment. This would give a valuable new insight into organelle preparations, and could be used to extrapolate values for organelle enrichment/depletion. A large number of Arabidopsis subcellular proteomes have been collated into SUBA, the SUBcellular localization database for Arabidopsis proteins (Heazlewood *et al*., 2005, 2007; Tanz *et al*., 2013; Hooper *et al*., 2017). To date, a third of Arabidopsis proteins have been experimentally localized to a subcellular compartment. With the recent development of SUBAcon (Hooper *et al*., 2014), a Bayesian algorithm to infer localization by probable consensus from experimental and predictive localization data, a resource is available to define the most probable single subcellular location for Arabidopsis proteins. These protein localization data are the product of over a decade's worth of intense proteomic analysis of organelles in Arabidopsis. In this study we utilize the efforts from these laboratories by incorporating organelle proteome data into the current *in silico* estimations of Arabidopsis protein abundance. We also add three new datasets containing enrichments of all the major subcellular compartments. From this we have generated a new abundance score for Arabidopsis proteins, termed the normalized protein abundance scale (NPAS), and have combined this with SUBAcon localizations to develop an interface called multiple marker abundance profiling (MMAP). The MMAP interface is available through the SUBA4 toolbox (http://suba.live) and can be used to estimate the subcellular composition of any list of protein identifications, even where no additional experimental data are available. By calculating the probability of identifying a protein from a given location, MMAP also delivers quantitative estimation of organelle enrichment. This approach paves the way for alternative methods for estimating protein abundance that could be applied to other species.

## Results

### A global protein abundance score (PAS) for Arabidopsis proteins observed by mass spectrometry

In 2012, 46 Arabidopsis proteomes containing spectral data from over 20 589 proteins were combined to make the first relative abundance estimate of Arabidopsis proteins in a theoretical whole‐organism proteome, housed at paxdb.org (Wang *et al*., 2012b, 2015). This collection was based on spectral data from tissue proteomes, and did not contain organelle proteomes. Consequently, not all proteins could be included, particularly the low‐abundance proteins that usually require extensive enrichment of certain subcellular regions before they can be detected, even on powerful mass‐spectrometers. Over 100 publications describing the proteomes of enriched subcellular regions, organelles and protein complexes are contained in SUBA, PPDB and AtChloro (Sun *et al*., 2009; Ferro *et al*., 2010; Hooper *et al*., 2017). These studies are an invaluable data resource for low‐abundance proteins, and would be a useful addition to an *in silico* PAS. However, data for these proteins can only be incorporated into an *in silico* scoring system if it is possible to appropriately scale data from enrichments into the tissue data. Therefore, the first challenges in expanding the current *in silico* PAS system were to curate all useable enrichment data and integrate these into the existing PaxDb score. Usable data from publications housed in SUBA4, PPDB and AtChloro (Table S1) were combined, delivering spectral data for 17 322 proteins. Even after combining all these data, coverage of some subcellular compartments was poor. For example, there are only two datasets (Dunkley *et al*., 2006; Nikolovski *et al*., 2012) describing an endoplasmic reticulum (ER) enrichment, compared with almost 20 plasma membrane (PM)‐related proteomes accumulated since 2004 (Hooper *et al*., 2017). Therefore, with the aim of equalizing coverage of the main subcellular compartments, a protoplast homogenate was separated along a linear density gradient. Fractions were selected that showed the best enrichment in each of the major subcellular compartments, including the cytosol and nucleus, for three biological replicates. Using protoplasts prevented analysis of the cell wall, but this region has been well covered in several recent proteomes, summarized in San Clemente and Jamet (2015). Peptides from newly acquired datasets, and published datasets for which peptide spectral matches were available, were then scored using the same method as used at paxdb.org (Wang *et al*., 2012a). Publications containing only protein‐level spectral data were also scored and included, as detailed in the Experimental Procedures. Scores were normalized by centring values for shared proteins on the PaxDb median (Figures S1 and S2), and scaled as detailed in the Experimental Procedures. This gave a PAS with standard deviation (log_10_‐space) for 23 191 proteins (Table S2), of which 2602 had not previously been scored. Normalization to the score total gave the NPAS. Subtracting or adding the exponentiated standard deviation values from PAS, then normalizing to the PAS total gave values for NPAS_min and NPAS_max (Table S2).

**Figure 1:**
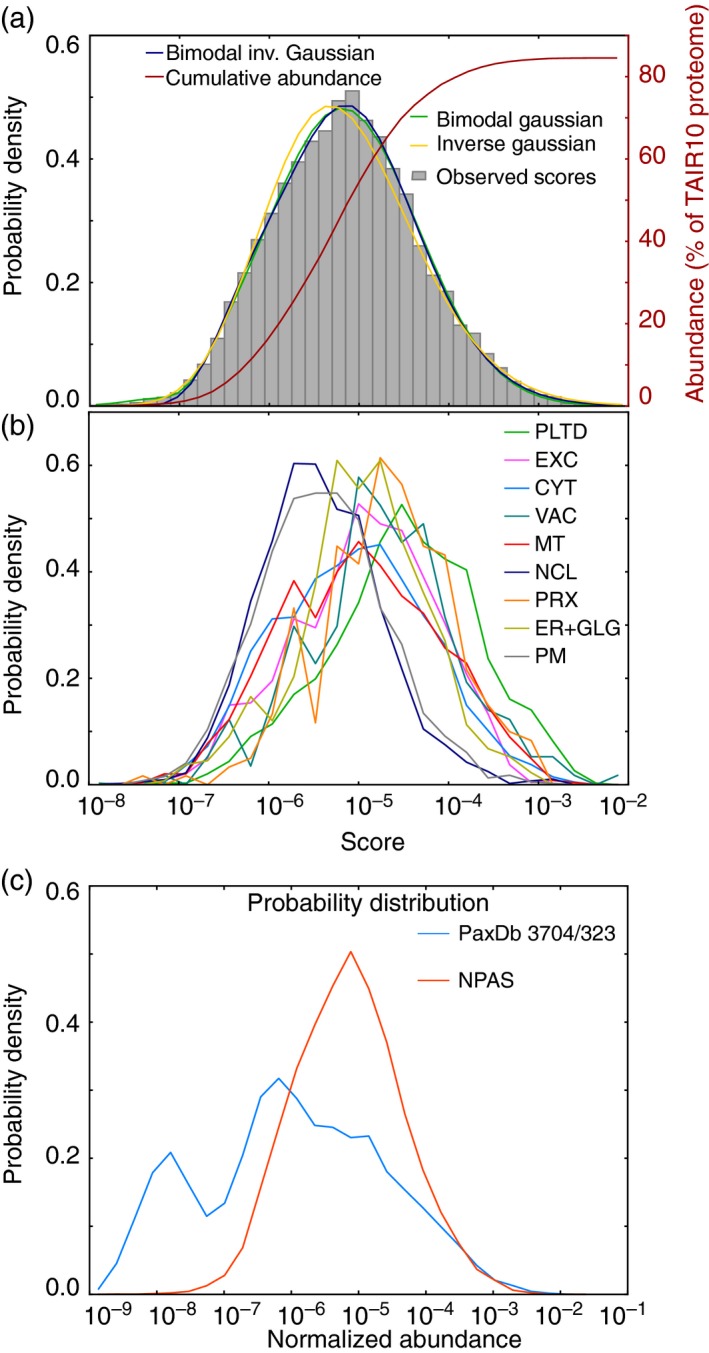
Normalized protein abundance scale (NPAS) distribution within the Arabidopsis proteome. A histogram showing the distribution of protein scores along the NPAS scale was plotted and compared with a range of commonly used probability distributions, including an inverse Gaussian distribution. Non‐linear least‐squares regression (Levenberg–Marquatt algorithm) was performed, and optimal best‐fit parameters for distribution were found using the Scientific Python module scipy.optimize.curve_fit. The cumulative number of scored proteins was plotted as a percentage of the TAIR10 Arabidopsis proteome (a). Proteins were assigned to locations using a collection of high‐confidence (HC) organelle markers (Table S3), as detailed in the subsequent results section, and the distribution of markers for each subcellular compartment occurring along the NPAS scale was plotted (b). A probability density histogram was used to compare NPAS distributions with abundance distributions for existing Arabidopsis abundance data from paxdb.org (c). Cyt, cytosol; ER, endoplasmic reticulum; EXC, extracellular; GLG, Golgi; MT, mitochondria; NCL, nucleus; PLTD, plastid/chloroplast; PRX, peroxisome; PM, plasma membrane; VAC, vacuole.

As NPAS was intended for comparison against *in vivo* data, it was important to establish whether the abundance distribution described by NPAS was representative of *in vivo* distributions. The statistical distribution of proteins has only been specifically investigated in mammalian cells. Results suggested an inverse Gaussian or Sichel distribution (Koziol *et al*., 2013). A somewhat similar distribution was anticipated, although an exact fit was unlikely given the physiological differences between Arabidopsis and mammalian cells. NPAS values for the Arabidopsis proteome closely fitted a bimodal inverse Gaussian distribution (Figure 1a). This fit was further investigated by examining the count distribution for individual subcellular compartments, using collections of high‐confidence (HC)‐markers (Table S3) generated for the main subcellular compartments, as detailed subsequently. The bimodal distribution was mainly attributable to higher‐scoring proteins in the plastid compared with other compartments (Figure 1b), indicating that the bimodality was a result of cellular differences between plants and mammals. NPAS was then compared with the original PaxDb scores, which covered 76% of the predicted Arabidopsis proteome (Wang *et al*., 2012a). NPAS distribution was considerably less bimodal than PaxDb scores, indicating more accurate protein scoring (Figure 1c), and NPAS increased the total Arabidopsis proteome coverage by almost 10% (Figure 1a). Plotting the number of expressed sequence tags associated with a gene against NPAS showed that, similar to previous reports (Greenbaum *et al*., 2003; Gry *et al*., 2009), a variable correlation exists between transcript and protein abundance (Figure S3a), confirming the requirement for an accurate *in silico* protein abundance estimate.

### Development of HC subcellular marker lists for Arabidopsis

**Figure 2:**
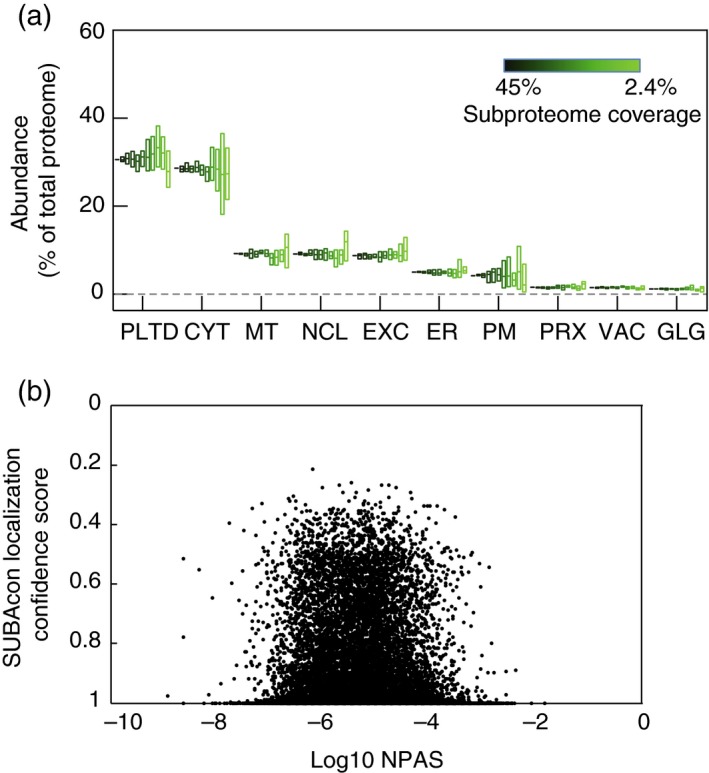
Analysis of high‐confidence (HC)‐marker collections. Variation in the proportional sizes of subcellular compartments estimated using proteomes of different sizes. HC‐marker sets were reduced from 45, 23, 18, 14, 9, 5, 2.4% coverage. Random reductions were performed six times and normalized protein abundance scale (NPAS) summed for each compartment proteome. Boxes represent value ranges, central horizontal bars show mean values (a). It was investigated whether, by selecting proteins with high localization confidence scores, a bias towards abundant proteins had been introduced into HC‐marker collections. Plotting NPAS against localization confidence values from Hooper *et al*. (2014) did not show any substantial bias (b). Abbreviations are as for Figure 1.

Using NPAS for estimating organelle composition required the development of improved organelle marker collections with which to categorize proteins into subcellular locations. It was desirable that marker collections were both accurate and extensive enough to capture the diversity of organelle proteomes. Also, collections needed to represent approximately the same proportion of each organelle proteome. This is challenging because an experimental survey of all Arabidopsis organelle proteome sizes has not yet been carried out. SUBAcon is an algorithm that integrates experimental evidence and computational prediction of a protein's subcellular location. Starting with proteome sizes derived from a mixture of plant, yeast and mammalian data (24, 27–30), SUBAcon attempts to assign a single location to every protein in the Arabidopsis proteome, using continuously updated experimental and predictive localization data. This way, SUBAcon provides the most comprehensive estimate of relative proteome sizes, to date, for the major subcellular destinations in Arabidopsis. Using unfiltered SUBAcon organelle proteome assignments gave extensive marker collections, but examining experimental metrics, such as the proportion of correctly‐localized fluorophore‐tagged proteins (FPs), often disagreed with consensus locations. SUBAcon is conservative; consequently, it often estimates a single location for proteins that, in reality, have multiple locations. Ideally, these multi‐localized proteins would not be included in marker collections. Therefore, rather than taking the entire SUBAcon proteomes, proteins were ranked by location confidence value (as calculated in Hooper *et al*., 2014), and only the most confident, singly‐localized proteins were included. The maximum proportion of a proteome that could be included, before compromising accuracy, was estimated by observing when the majority of SUBAcon location predictions were no longer supported by the available experimental (FP and LC‐MS/MS) data. This was first found to occur at between 40 and 45% of the ranked SUBAcon cytosolic proteome. Therefore, only the top‐ranked 45% of each organelle proteome was used as markers. The SUBAcon algorithm cannot easily distinguish data‐rich, multi‐localized proteins. Therefore, the top 45% of proteins for each organelle were manually edited, and proteins that were rich in predictive and experimental data, but poor in data agreement, were replaced with proteins that were just below the 45% threshold, but were still supported by experimental localization data as being genuine organelle residents. Subcellular localizations throughout each organelle marker list were then manually checked for consistent support from experimental or predictive data. This yielded between 133 (peroxisome) and 3274 (nucleus) marker proteins per organelle, referred to as the HC‐marker collection (Tables 1 and S3). At 45%, the variance in estimating the size of each organelle proteome was considerably reduced (Figure 2a), indicating that this value provided sufficient representation of each proteome. Plotting localization confidence values against NPAS for markers in Table S2 revealed little correlation between confidence value and abundance (Figure 2b), indicating that the HC selection process had not biased the representative protein abundance. Marker list accuracy was assayed by examining the proportion of proteins that had FP localization data, and that localized as anticipated when the consensus localization was taken from the FP localization data. On average, 80% of green fluorescent protein (GFP) consensus localizations were correct (Table 1), indicating a high level of marker accuracy. Only 40% of extracellular localizations were correct, but FP localization data were available only for 5% of the extracellular HC‐markers. Overall, these data showed that HC‐markers lists, comprising 45% of each predicted proteome, gave a good balance of coverage and accuracy for subcellular assignments. HC‐markers (Table S3) were therefore used to assess shotgun proteomics data for the remainder of the study.

**Table 1:** Summary of HC‐marker collections

Location	Number of HC‐markers (Table S2)	Estimated proteome size	Proteome coverage (%)	GFP localization (%)	Correct GFP localization (%)
CYT	2512	5587	45	15	74
ER	323	716	45	40	82
EXC	1495	3320	45	5	40
GLG	239	524	45	41	92
MT	1073	2383	45	22	83
NCL	3274	7274	45	14	87
PRX	135	295	45	61	85
PLTD	1437	3192	45	20	80
PM	1193	2649	45	22	85
VAC	213	469	45	46	84

After ranking by location confidence (Hooper *et al*., 2014) and manually editing data‐rich, multi‐localized proteins from collections, the top 45% of each SUBAcon subcellular proteome (Hooper *et al*., 2014) was used to assign proteins to subcellular locations. Proteome sizes were estimated previously by Hooper *et al*. (2017). Marker collections are detailed in Table S3. The proportion of markers from each HC organelle collection with associated FP localization data was compared with the number of correctly‐localized FP markers within each collection. ‘Correct localization’ was defined as the majority consensus localization for all confocal data, housed at Hooper *et al*. (2017), for each tagged protein.

CYT, cytosol; ER, endoplasmic reticulum; EXC, extracellular; GFP, green fluorescent protein; GLG, Golgi; HC, high‐confidence; MT, mitochondria; NCL, nucleus; PLTD, plastid/chloroplast; PM, plasma membrane; PRX, peroxisome; VAC, vacuole.

### Profiling organelles by NPAS, HC‐markers and SRM

**Figure 3:**
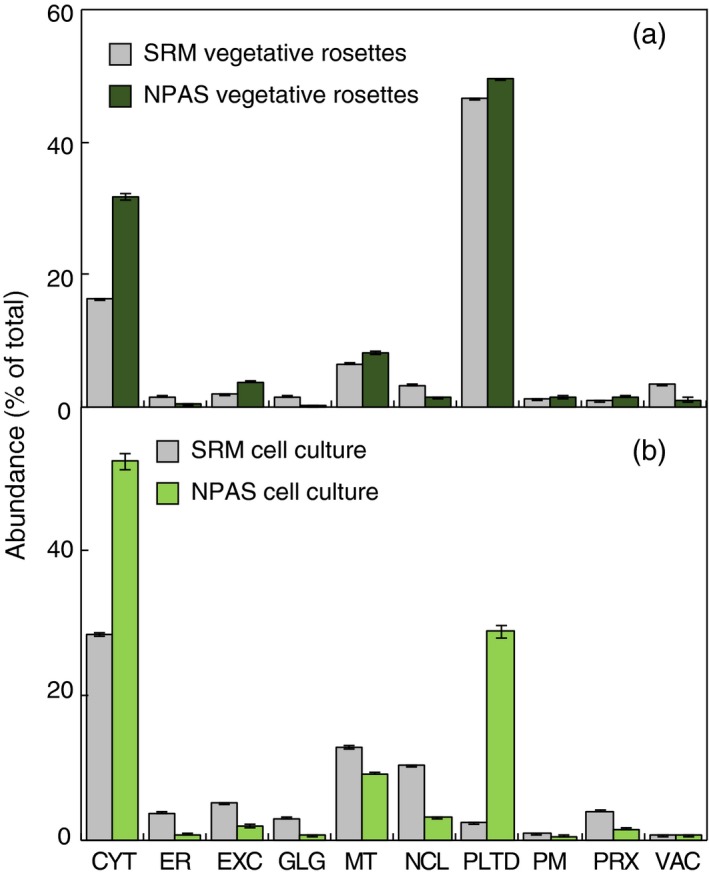
Assessment of selected reaction monitoring (SRM) as an independent estimate of relative subcellular compartment abundance. The ability of SRM, an high‐confidence (HC)‐marker‐independent technique, to deliver broadly comparable estimates of subcellular composition to normalized protein abundance scale (NPAS) was tested. The performance of SRM and NPAS was compared in five independent replicates from vegetative rosettes (a) or cell‐suspension culture (CSC; b). Protein identifications from shotgun analyses were assigned locations using HC‐markers in Table S3. NPAS was summed for each location and expressed as a proportion of the total for all locations. Fragment ion intensities from SRM markers (Table S4) were averaged per location and presented as a percentage of the total fragment ion intensity for comparison with NPAS. Abbreviations are as for Figure 1.

**Figure 4:**
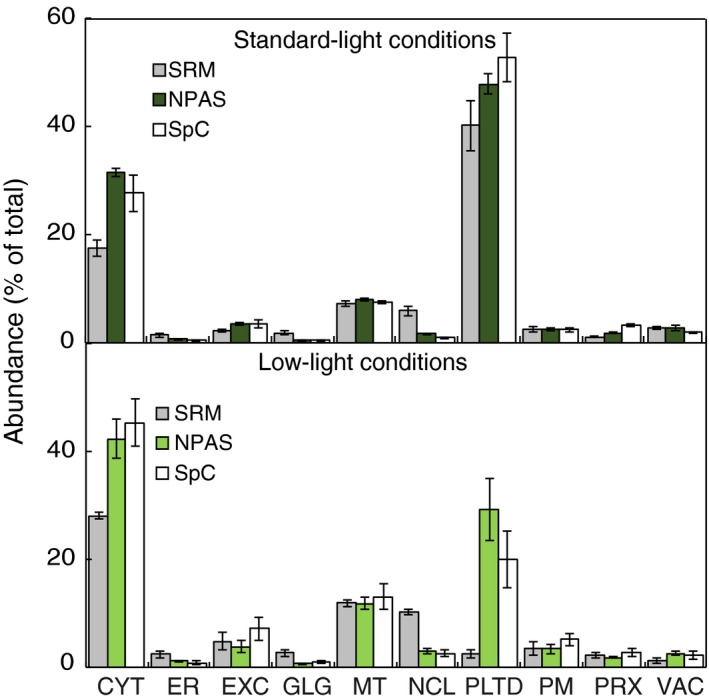
Estimation of subcellular composition using normalized protein abundance scale (NPAS), compared with estimates from spectral counting (SpC) and selected reaction monitoring (SRM), in different issues and growth conditions. Proteins were extracted from vegetative rosettes, reproductive rosettes, stem 2nd internode, cauline leaves, green siliques and seedlings grown in long‐day conditions (a). Protein extracts were subject to analysis by SRM or shotgun LC‐MS/MS. Proteins identified from shotgun analysis were assigned to subcellular locations using high‐confidence (HC)‐markers (Table S3), and either NPAS or SpC were summed for each location. This was repeated for plant material grown under lower light conditions [cell‐suspension culture (CSC), roots and etiolated seedlings; b]. Results were expressed as a percentage of location totals. Error bars show s.e. for *n* = 5 (long‐day conditions) or *n* = 3 (low‐light conditions).

Having established a method for estimating protein abundance *in silico*, and suitable marker lists for assigning protein location, the combined ability of NPAS and HC‐markers to detect changes in subcellular sample composition was tested. SRM was chosen as an alternative method for validating NPAS measurements of organelle abundance. SRM is a targeted proteomics technique that detects peptides of interest by focusing on a limited number of pre‐determined targets. By using several carefully chosen protein markers from each organelle, SRM can be used to estimate relative organelle abundance (Parsons and Heazlewood, 2015). Measurements by SRM do not depend on the use of HC‐markers and avoid the stochastic element of shotgun proteomics measurements. As NPAS scores are both based on shotgun data and dependent on HC‐markers, SRM was an appropriate choice of validation. A systematic set of SRM organelle markers has not yet been developed in plants. Therefore, an exhaustive survey of transitions from multiple abundant organelle marker proteins was assessed by mass spectrometry of whole‐cell protein lysates until a collection of reliable markers was identified. For the 10 defined subcellular compartments, a minimum criterion of three marker proteins was accomplished, except for the vacuole where only two could be reliably used (Table S4). Transitions and retention times were verified with stable isotope‐labelled internal peptide standards (Table S4). SRM was evaluated as a comparative measure against NPAS by examining the ability of both SRM and NPAS to report differences in relative organelle abundance between rosettes and cell‐suspension culture (CSC). Validation was limited to the 10 main subcellular compartments as the diverse population of proteins not assigned a location by HC‐markers could not be represented within a limited set of SRM markers. Nevertheless, this provided adequate data to show whether SRM could detect sufficient changes to be used as a validation method. SRM measurements of relative organelle abundance were consistent over five independent replicates (Figure 3), showing it to be a technically robust method for comparison. Organelle profile differences were observed in rosette leaves, most notably in the cytosol, but profiles were of broadly sufficient similarity that SRM was deemed to be a suitable validation technique (Figure 3a). When tested in CSC, organelles profiles differed more than in rosettes (Figure 3b). Extracellular and peroxisomal proteins were over‐scored by NPAS, over‐scoring of the nucleus was greater than in rosettes, and the plastid was reported at dramatically lower values (Figure 3b). Therefore, although SRM was clearly capable of providing a comparative measure (Figure 3a), at least one of the techniques was failing to report accurately in certain contexts. Both SRM and NPAS were new approaches to measuring organelle abundance, so it was not clear from which technique the discrepancy originated. Experiments were therefore repeated in a wider range of tissues and growth conditions, and a third measure of organelle abundance, spectral counting (SpC), was introduced (Lundgren *et al*., 2010). As with NPAS, SpC was used in conjunction with HC‐markers to estimate organelle abundance. When assessing the usefulness of SRM as a validation for NPAS, it had become evident that either NPAS or SRM reported plastid content less accurately in CSC than rosettes (Figure 3), leading to the suggestion that scoring sensitivity could be affected by light levels. Therefore, during the three‐way comparison between NPAS, SRM and SpC (Figure 4), Arabidopsis samples were grouped into standard‐light (vegetative rosettes, reproductive rosettes, cauline leaves, stem internode, green silique, 7‐day‐old seedlings) and low‐light growth conditions (CSC, roots, etiolated seedlings). The overall findings from the three‐way comparison were that NPAS accurately reported changes in organelle abundance over a range of different plant material and growth conditions. In the standard‐light‐grown group NPAS was always similar to at least one of the other two techniques, never reporting outlying values (Figure 4a). The discrepancy in relative cytosolic values observed in Figure 3 therefore appeared to stem from SRM, not NPAS. NPAS also performed well in low‐light‐grown material, excepting plastids. Here NPAS reported significantly higher plastid levels than either SRM or SpC (Figure 4b). Examining material from individual sources of plant material showed this was primarily due to the reporting of unusually high plastid levels by NPAS in CSC (Figure S4a). SRM reported plastid levels as dramatically lower than SpC or NPAS (Figure 4b), showing that the discrepancy between plastid values in Figure 3b stemmed from both techniques, but the overwhelming contribution came from SRM.

The three‐way comparison also presented an opportunity to examine the viability of SRM as a stand‐alone technique for estimating subcellular composition. SRM is an appealing alternative to immunoblotting, given the time and resources needed to develop antibodies in new species, compared with the expanding number of research species with sequenced genomes in plant sciences. Although SRM appeared to report relative organelle abundance less accurately than NPAS, it did give broadly similar organelle abundance profiles – the major exception being plastid levels in material grown in low‐light conditions (Figure 4b). Examining the response of individual SRM target peptides for the plastid showed that all plastid peptide signal intensities decreased in response to low‐light conditions, but this was most obvious for markers directly related to photosynthesis (Table S5). Not all plastid proteins identified in shotgun data exhibited such strong responses, indicating that light‐responsive proteins were over‐represented amongst the chosen SRM targets. SRM had also over‐estimated the Golgi and nucleus compared with NPAS and SpC (Figures 3 and 4). Golgi and nuclear SRM targets all showed similar changes in intensity, indicating that the three target proteins likely represented above‐average expression levels for these two organelles. However, highlighted differences aside, results showed that NPAS delivered excellent estimation of subcellular composition in a wide range of contexts. SRM performed well, except when reporting plastid levels and, to a lesser extent, cytosolic and nuclear levels.

### Estimating sample composition and organelle enrichment using the MMAP tool

**Figure 5:**
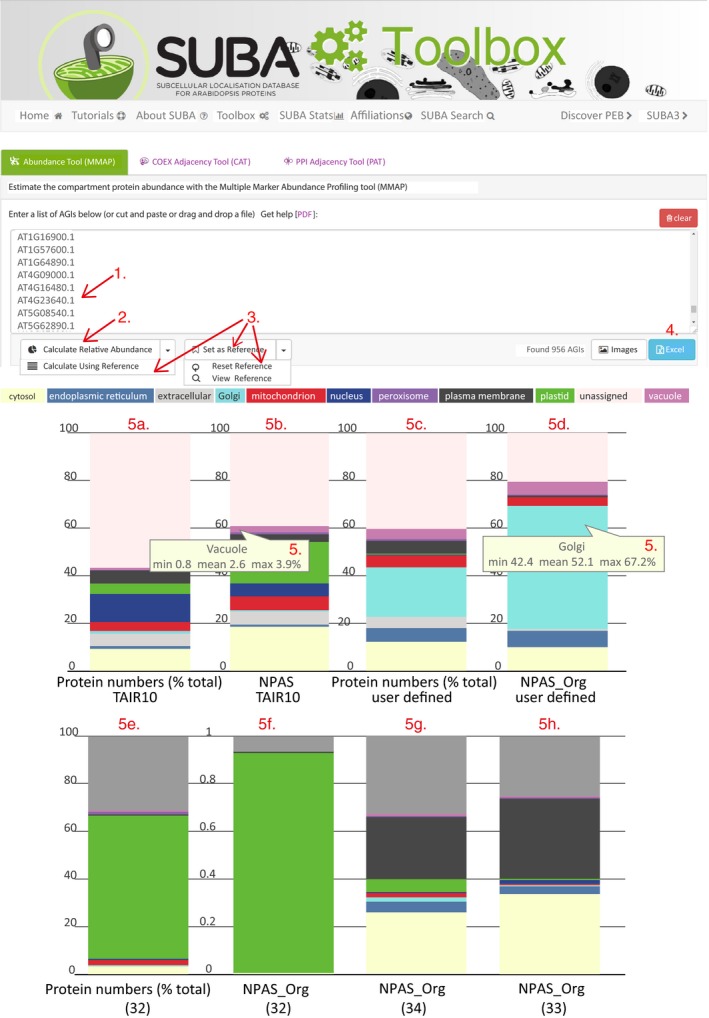
Demonstration and use of the multiple marker abundance profiling (MMAP) online tool using organelle proteomes. Using the Golgi proteome from Parsons *et al*. (2012), the features of the MMAP online tool are introduced. First, proteins identifiers are pasted into the input box (arrow 1) and the output is calculated (arrow 2). If required, the reference proteome (as an alternative to TAIR 10) is set, or reset (arrow 3). Data are visualized in stacked bar graphs (a–d) or downloaded (arrow 4). Data are presented as absolute protein numbers (a, b) and compared against normalized protein abundance scale (NPAS) for the TAIR10 proteome (d), or NPAS output after scaling NPAS sums to account for organelle enrichment or depletion. The analytical capacity of MMAP is further demonstrated using the chloroplast proteome (e, f) from Zybailov *et al*. (2008) and comparing the scaled NPAS output of two methods for isolation of plasma membrane (PM) vesicles (g, h) from Elmore *et al*. (2012) and de Michele *et al*. (2016).

Analysing sample composition using a combination of NPAS and HC‐markers could be useful to many researchers wanting a preliminary assessment of experimental data before committing to further experimentation. Therefore, the MMAP tool, based on the above methods, has been made available at SUBA4 (http://suba.live/) in the ToolBox section (Figure 5). The features of MMAP are demonstrated using data from a previously published Golgi proteome (Parsons *et al*., 2012), a chloroplast proteome (Zybailov *et al*., 2008), and two different approaches used to isolated PM vesicles (Elmore *et al*., 2012; de Michele *et al*., 2016). The list of protein identifications from the Golgi proteome was downloaded from SUBA4 and pasted into the MMAP input box (Figure 5, arrow 1). Clicking ‘calculate relative abundance’ (Figure 5, arrow 2) generates four stacked bar graphs showing the proportion of proteins assigned to subcellular compartments (Figure 5a–d), which, unlike the SRM comparison (Figures 3 and 4), now includes an unassigned category. The default reference dataset is TAIR10, but pasting a list of AGIs into the input box and clicking ‘set reference’ sets the pasted list as the reference data until ‘reset reference’ is chosen (Figure 5, arrows 3). During the MMAP process, the whole Arabidopsis proteome is first grouped by HC‐markers, with Figure 5a showing absolute protein numbers and Figure 5b showing protein abundance, as scored by NPAS. Figure 5a and b is compared with Figure 5c and d. Figure 5c shows absolute protein numbers assigned to organelles in the user‐submitted dataset, and Figure 5d shows organelle abundance in the user‐submitted dataset. User‐submitted organelle abundance cannot be directly measured by summed NPAS scores as these are fixed, standard values that do not describe protein abundance after enrichment or depletion of organelles. Instead, organelle abundance in user‐submitted samples is described using NPAS_Org. NPAS_Org is a probabilistic organelle abundance value calculated using an abundance‐scaling factor. The abundance‐scaling factor calculates organelle depletion or enrichment according to the probability of observation in the Arabidopsis proteome, and the observed frequency in the user‐submitted sample, as detailed in the Experimental Procedures. Multiplying the original organelle abundance estimates by the abundance‐scaling factor gives NPAS_Org. Using the log10‐standard deviation values associated with PAS (Table S3) allows the error associated with compartment enrichment to be calculated. This can be visualized by hovering over the NPAS and NPAS_Org values shown in the stacked bar graphs (Figure 5a–d, arrow 5). Output data can be downloaded in as comma‐separated values (.csv) format (Figure 5, arrow 4). The abundance‐scaling factor was validated by showing that changes in abundance‐scaling factors directly corresponded to the changes in organelle abundance measured by SpC (Figure S6). The analytical capacity of MMAP was demonstrated by comparing an MMAP analysis of progressive Golgi enrichment (Table 2) with an equivalent immunoblotting analysis (fig. 2 in Parsons *et al*., 2012). Starting from CSC, Golgi membranes were first enriched by density‐gradient centrifugation (Table 2, ‘pre‐FFE’), then by free‐flow electrophoresis (Table 2, ‘post‐FFE’). Shotgun data were available for all three stages (PRIDE Project https://doi.org/10.6019/pxd005408), so enrichment could be analysed by MMAP, with the CSC proteome set as the reference. Comparing MMAP with immunoblot results showed agreement for the larger, more obvious changes in subcellular composition. For example, immunoblots reported a large increase in ER between CSC and pre‐FFE samples and a small increase in Golgi (Parsons *et al*., 2012). MMAP reported similar changes in ER and Golgi (Table 2), but now quantitative estimates could be placed on these increases (Table 2). Where changes in organelle abundance were subtler, MMAP and immunoblot measurements agreed less. For example, MMAP reported little change in cytosolic or plastid levels between CSC and pre‐FFE samples (Table 2), but immunoblots showed a decrease (Parsons *et al*., 2012). Although MMAP and immunoblots agreed on a sizable Golgi enrichment in post‐FFE samples, immunoblots showed an appreciable decrease in the ER and increase in mitochondria compared with the CSC starting material (Parsons *et al*., 2012), but MMAP reported a proportional ER increase and mitochondrial decrease (Table 2). Electron micrographs of successive enrichment stages (Parsons *et al*., 2012) showed better agreement with the MMAP analysis of post‐FFE samples, as images contained no visible mitochondria, indicating that MMAP analyses were more biologically representative of organelle contamination than immunoblotting.

**Table 2:** Subcellular sample composition during successive stages of Golgi enrichment

Location	NPAS TAIR10	NPAS_Org CSC	NPAS_Org Pre‐FFE	Fold change from CSC	NPAS_Org post‐FFE	Fold change from CSC
CYT	0.186	0.1546	0.1445	1	0.0204	0
ER	0.009	0.0008	0.0521	66	0.0147	19
EXC	0.053	0.0028	0.0030	1	0.0014	0
GLG	0.007	0.0013	0.0087	7	0.1101	83
MT	0.056	0.0240	0.0046	0	0.0072	0
NCL	0.056	0.0046	0.0018	0	0.0001	0
PM	0.174	0.0365	0.0009	0	0.0000	0
PTD	0.031	0.0007	0.0035	5	0.0020	3
PRX	0.010	0.0083	0.0056	1	0.0007	0
VAC	0.026	0.0003	0.0026	10	0.0113	42
Unassigned	0.391	0.0940	0.0715	1	0.0435	0

Using the CSC proteome as the reference dataset, subcellular composition was examined during successive stages of organelle enrichment using MMAP. Endomembranes were first enriched on a step‐gradient according to Parsons *et al*. (2012) (pre‐FFE), then Golgi membranes were extracted using free‐flow electrophoresis (post‐FFE). Post‐FFE data comprise the Golgi proteome of Parsons *et al*. (2012), as downloaded from SUBA (suba3.plantenergy.uwa.edu.au). ‘NPAS TAIR10’ describes summed NPAS for subcellular locations in the whole Arabidopsis proteome. ‘NPAS_Org’ values refer to the calculated relative abundance of subcellular locations, after applying the abundance‐scaling factor as described in the Experimental Procedures.

CSC, cell‐suspension culture; CYT, cytosol; ER, endoplasmic reticulum; EXC, extracellular; FFE, free‐flow electrophoresis; GLG, Golgi; MT, mitochondria; NCL, nucleus; NPAS, normalized protein abundance scale; PLTD, plastid/chloroplast; PRX, peroxisome; PM, plasma membrane; VAC, vacuole.

Golgi stacks are small and low in number compared with other organelles, so the performance of MMAP was contrasted to a chloroplast proteome from leaves (Figure 5e and f). A large number of proteins were identified from the proteome of Zybailov and co‐workers (Zybailov *et al*., 2008; Figure 5e). Nevertheless, NPAS_Org revealed a greater enrichment for plastid proteins, and decreased contribution from other organelles than had been apparent using protein numbers alone (Figure 5e and f). The rapid methodological comparisons achievable using MMAP were demonstrated by comparing two PM isolations (Elmore *et al*., 2012; de Michele *et al*., 2016). Both methods had enriched for PM vesicles using two‐phase partitioning, but one (de Michele *et al*., 2016) used FFE to further purify phase‐partitioned PM vesicles. Comparing the NPAS_Org output (Figure 5g and h) showed that the additional use of FFE decreased contamination from the plastid, Golgi and mitochondria. FFE had little effect on reducing ER or contamination, and the relative cytosolic contribution increased, but the effects of this extra purification step were overall positive and could be quickly visualized using MMAP.

### Analysis of tissue proteomes using MMAP

**Figure 6:**
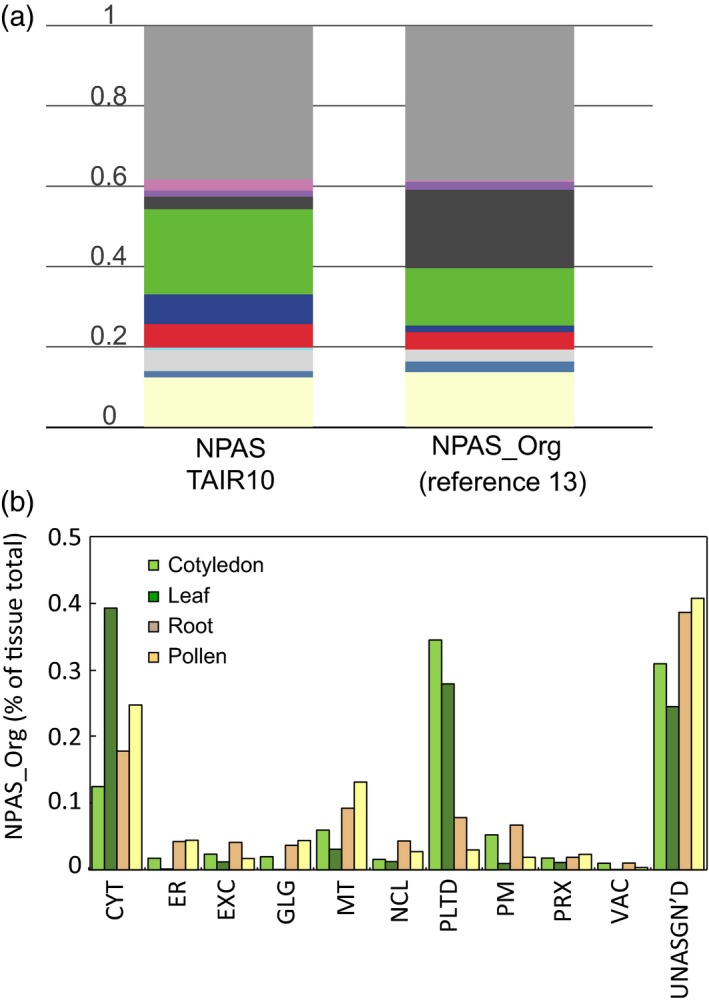
Applicability of multiple marker abundance profiling (MMAP) to analysing tissue proteomes. The use of MMAP in high‐throughput, rapid analysis of the subcellular composition of different tissues proteomes was demonstrated by analysis of the plasmodesmata proteome from Fernandez‐Calvino *et al*. (2011). Stacked bar graphs show the NPAS_Org output from MMAP, which is compared with summed normalized protein abundance scale (NPAS) for the total Arabidopsis proteome (a). The colour scale was the same as Figure 5. A selection of tissue proteomes (Grobei *et al*., 2009; Piques *et al*., 2009; Baerenfaller *et al*., 2011) were analysed using MMAP. NPAS_Org output is compared in (b).

The applicability of MMAP is not only restricted to organelle enrichments where large changes in sample composition are expected, but is also useful for analysing the composition of tissue proteomes. Figure 6a shows an MMAP analysis of the plasmodesmata proteome (Fernandez‐Calvino *et al*., 2011). A decrease in Golgi, vacuole, peroxisome, mitochondria and cytosol levels compared with standard values was reported by MMAP, along with an appreciable increase in the PM and ER (Figure 6a). In Fernandez‐Calvino *et al*. (2011), immunoblotting with anti‐PM marker antibodies reported low levels of PM in plasmodesmata, leading the authors to suggest that the altered protein composition of specialized PM domains may have affected immunoblotting results (Fernandez‐Calvino *et al*., 2011). This shows the superior analytical capacity of MMAP, which uses many hundreds of subcellular markers. MMAP can also be used to generate an overview of relative organelle abundance for different tissue proteomes, the same tissue following a treatment or environmental stimuli, or in mutant proteome phenotyping, giving an insight into how organelle proportions relate to tissue function. We demonstrate this by comparing proteomes from four different tissues; cotyledons, leaf, root and pollen (Grobei *et al*., 2009; Piques *et al*., 2009; Baerenfaller *et al*., 2011). Changing organelle proportions reflected tissue specialization; plastid levels were highest in green tissue, and ER, Golgi and mitochondrial levels were highest in pollen and roots, i.e. tissue associated with tip growth (Figure 6b). Both cotyledons and roots had a large proportion of PM proteins, likely reflecting high metabolic exchange in these tissues. The leaf proteome had a proportionally large cytosol which, after downloading results and examining the cytosolic content, appeared to be the result of many high‐scoring cytosolic ribosomal proteins. The extracellular component was relatively high in roots. Closer examination revealed many of the high‐scoring proteins unique to the root proteome were peroxidases, a family of proteins known to be involved in root cell expansion (Dunand *et al*., 2007).

## Discussion

This study describes the development of an *in silic*o abundance score (NPAS) for Arabidopsis proteins. Using spectral data from newly‐acquired subcellular proteomic datasets and previously published organelle proteomes, we assigned standard abundance values to 85% of 27 416 proteins, compared with 76% in an earlier curation of Arabidopsis protein abundances. HC‐markers were defined for subcellular locations (Table S3) and combined with NPAS (Table S2), so that abundance of organelles could be estimated from *in silico* protein abundance values (Figure 4). This was further extended into an online tool (MMAP), which gives instant analysis of subcellular composition compared with a reference dataset (Figure 5). The NPAS_Org feature in MMAP permits assessment of changing organelle ratios between samples and references, by calculating the probable enrichment or depletion of organelles (Figures 5 and S5). MMAP requires only a list of protein identifiers as input, so data from a broad range of sources can be analysed. For example, output from contemporary methods can be compared with older, published methods for which spectral data may not be available. MMAP also provides a useful template for developing a similar approach in other species. The number of plant research species has expanded rapidly as interest in establishing a secure food supply in a changing climate has increased and, although antibody availability has also increased, it cannot match pace. MMAP can potentially solve the requirement for antibodies when investigating sample or tissue composition in other species. For some species, the cumulative bank of proteomics data is reaching the point at which the MMAP approach could be copied (Hooper *et al*., 2016; Rathi *et al*., 2016). For other species, submitting a list of close Arabidopsis homologues to MMAP could yield sufficiently useful information.

Multiple marker abundance profiling is dependent on the inclusivity and accuracy of the HC‐marker collections (Table S3). Marker accuracy was validated using previously published FP localizations (Table 1). Organelle abundance estimations were validated by comparison with SpC and SRM, the latter being independent of HC‐markers (Figure 4). The high GFP accuracy and large number of markers (Table 1) show that a balance between accuracy and inclusivity was successfully achieved. However, when estimates of relative organelle abundance were compared with estimates using SRM, Golgi and nuclear levels were consistently under‐estimated. Given the length of the nuclear HC‐collection (3274 proteins), it is unlikely this resulted from an underestimate of the nuclear proteome size. Rather, above‐average abundant proteins may have been chosen as nuclear SRM makers, as prior to NPAS estimating the abundance of a protein marker relative to other residents of the same location was difficult. Finding a SRM marker close to the NPAS average for each compartment could improve SRM organelle markers in the future. Another unexpected inconsistency observed during comparison of SRM, NPAS and SpC was that NPAS overestimated the plastid content in CSCs and, to a lesser extent, roots (Figures 4b, and S4a and b). Estimates were greater than those from SpC, even though both techniques depended on HC‐markers (Figure 4b). This pointed to a plastid‐specific consequence of estimating organelle size using NPAS in specific contexts. A possible explanation is related to the source of much of the spectral data used to generate NPAS. The cell line contains recognizable chloroplasts (Parsons *et al*., 2012), and contains many chloroplast proteins that are abundant in photosynthetic tissues. The data behind NPAS were derived mostly from photosynthetic tissue, so if the same proteins were present as in photosynthetic tissues, but were at appreciably lower abundances in the cell culture, NPAS would confer an overly high score onto these proteins. This limits the analytical scope of NPAS in certain, specific contexts but, as demonstrated in Figure 4, NPAS nevertheless reports relative organelle abundance reliably over a wide range of conditions.

Selected reaction monitoring and NPAS estimates of plastid abundance were very different in samples grown under low‐light conditions. A decrease in signal intensity was observed for all SRM targets (Figure 4b; Table S5), implying widespread light‐regulated protein expression amongst the SRM targets selected. Compared with transcriptomic data, little data are available showing response to stimuli at the protein level, so predicting SRM target response was difficult. Shotgun analysis of tissues recorded a diverse population of plastid‐localized proteins in low‐light‐grown samples, compared with samples derived from standard‐light conditions. Many of the plastid proteins thus identified showed no decrease in response to low‐light conditions, but the chosen plastid SRM markers could not report this. This highlights a key advantage of MMAP – that by using a large number of subcellular markers, changes in proteome composition can be accounted for when estimating organelle abundance, despite changes in the organelle proteome composition. This point was well illustrated by the plasmodesmata proteome (Figure 6a), where localized changes in protein composition of the PM prevented its detection by immunoblotting, although both gene ontology annotations (Fernandez‐Calvino *et al*., 2011) and MMAP (Figure 6a) showed an appreciable enrichment of PM proteins.

A useful feature of MMAP is the abundance‐scaling factor, which allows users to monitor changes in abundance after enrichment or depletion or organelles compared with TAIR10, or a user‐defined reference set. NPAS_Org can only be inferred from protein numbers in the user‐submitted dataset; protein abundance in a new dataset cannot be described using NPAS as this describes fixed, *in silico* values. In smaller datasets of about 2000 proteins, the identified proteins tend to lie within a relatively narrow abundance range compared with actual cellular ranges. This means that inferring protein abundance based on protein numbers gives a reasonably accurate interpretation of organelle abundance. MMAP is designed for use with single shotgun experiments, so this impacts little on results, but when tens of thousands of proteins over a large dynamic range are queried an effect is observed. For this reason, querying the entirety of TAIR10 does not return exactly the same NPAS and NPAS_Org values. Where organelle abundances were estimated from single shotgun experiments, a close match between the abundance‐scaling factor and organelle abundance was apparent for most organelles (Figure S6). These results show that despite this limitation, the abundance‐scaling factor provides a good means of estimating compartment abundance in experimental shotgun data.

Cross‐validating SpC and NPAS data with SRM allowed the potential for development of SRM as an alternative to immunoblotting to be assessed. Our thorough methodological comparison shows that the proteins chosen in this study provide an adequate preliminary suite of subcellular SRM markers, and highlights required improvements. As described, all plastid targets exhibited light‐dependent responses, which had not been anticipated from gene expression data. If the plastid markers described in Table S4 were expanded to include plastid targets consistently represented in proteomes of non‐photosynthetic tissues, then a suite of markers that accurately reported plastid abundance in a greater range of tissues could be produced. With the hindsight offered by NPAS, selecting protein markers with NPAS values distributed evenly across the compartment score range would also further improve representation by marker proteins.

In summary, we have developed a method for accurately estimating the subcellular composition of samples over a wide range of experimental conditions, using only a list of protein identifiers as the input, and we have produced an initial suite of organelle markers for targeted proteomic analysis. The latter contributes to ongoing efforts (Fan *et al*., 2012) in Arabidopsis, and provides a template for development in other species with sequence genomes but few available antibodies against organelle markers. The former will be very useful to researchers wishing to conduct quick, high‐throughput surveys of samples without committing to fully quantitative proteomics, and also offers options for analyses in other species.

## Experimental Procedures

### Plant material

*Arabidopsis thaliana* (L.) Heynh. Columbia‐0 (Col‐0) was obtained from the Arabidopsis Biological Resource Center. Plants were grown under long‐day conditions [16 h of fluorescent light (120 μmol m^−2^ s^−1^) at 22°C and 60% relative humidity (RH)/8 h of dark at 22°C and 60% RH]. The 4‐week rosette samples were harvested prior to bolting. CSCs were grown as previously described (Parsons *et al*., 2012). Seedlings were grown on MS agar. Etiolated seedlings were exposed to 24 h light then grown in darkness. Standard‐light‐grown samples comprised the following samples and post‐germination times: green seedlings (7 days); vegetative rosettes (4 weeks); green siliques (6 weeks); cauline leaf (8 weeks); 2nd stem internode (8 weeks); reproductive rosettes (8 weeks). Low‐light samples comprised the following: CSC (7 days post‐splitting); roots (6 weeks); etiolated seedlings (7 days).

### Protein extraction and sample preparation

Plant material was freeze‐dried and homogenized in a ball‐mill for 3 min at 30 Hz (Retsch). Protein was extracted with 125 mm Tris‐HCl, 7% (w/v) sodium dodecyl sulphate, 10% (v/v) β‐mercaptoethanol and plant protease inhibitor cocktail (Sigma Aldrich), precipitated by methanol‐chloroform water, resuspended in 8 m urea pH 8.0, reduced in 25 mm dithiothreitol (DTT), alkylated in 50 mm iodoacetamide and digested overnight at 37°C at a 1:10 trypsin:protein ratio after dilution to 1 m urea and adjustment to pH 8. Peptides were purified and concentrated using a C_18_ solid‐phase extraction procedure (Parsons *et al*., 2012).

### Data‐dependent acquisition by tandem mass spectrometry

For CSCs and 4‐week rosette samples (five replicates each), MS/MS data were acquired from about 1 μg peptides with a nano‐ESI‐Q‐TOF system (TripleTOF^®^ 5600 System, SCIEX) coupled to an Eksigent nano LC system (SCIEX). Peptides were separated on a Pepmap100 μ‐guard column (Dionex) via a Famos Autosampler (Dionex) and washed for 10 min with Buffer A (2% acetonitrile, 0.1% formic acid) flowing at 15 μl min^−1^. Peptides were eluted onto an Acclaim Pepmap100 C18 column (75 μm × 150 mm, 300 nl min^−1^ flow rate; Dionex) and into the TripleTOF 5600 via a gradient consisting of initial starting condition of 5% buffer B (98% acetonitrile, 0.1% formic acid) increasing B to 35% B over 60 min. Subsequently, B was increased to 90% over 3 min and held for 15 min followed by a ramp back down to 5% B over 3 min where it was held for 15 min to re‐equilibrate the column to the original condition. Peptides were introduced to the mass spectrometer via a Nanospray III source (SCIEX) with a nano‐tip emitter (New Objective) operating in positive‐ion mode (2400 V). The data were acquired with Analyst TF 1.5.1 operating in information‐dependent acquisition mode, whereby after a 250‐msec scan the 20 most intense ions (charge states 2–5) within 400–1600 m/z mass range above a threshold of 150 counts were selected for MS/MS analysis. MS/MS spectra were collected using TOF Resolution Mode: High Resolution with the quadrupole set to UNIT resolution and rolling collision energy to optimize fragmentation. MS/MS spectra were scanned from 100 to 1600 m/z, and were collected for a total accumulation time of 50 ms. selected precursor ions were excluded for 16 sec following MS/MS acquisition. The raw data were processed with the ProteinPilot Software package v.4.0 (SCIEX) and matched with the Paragon Algorithm against Arabidopsis proteins (TAIR10; Lamesch *et al*., 2012). The Paragon Method (Shilov *et al*., 2007) employed standard settings with the instrument set as ‘TripleTOF 5600’ resulting in initial search parameters of 0.05 Da (MS) and 0.1 Da (MS/MS). The detected protein threshold was set at 99% [Unused ProtScore (Conf) > 2.0] and a Thorough ID was applied for the Search Effort. The data processing and matching by ProteinPilot results in recalibration of data, which were subsequently exported as MGF Peaklist(s) for HC‐data matching. These raw data for the whole plant (*n* = 3) and CSCs (*n* = 3) are available at PRIDE (Project https://doi.org/10.6019/pxd005408).

For Arabidopsis low/high‐light samples, analysis was undertaken with about 1 μg protein and performed with a Q‐Exactive+ (Thermo Fisher Scientific) with a nanoACQUITY UltraPerformance LC system (Waters), incorporating a C_18_ reverse phase column (Waters; 100 μm × 100 mm, 1.7 μm particle, BEH130C18, column temperature 40°C). Peptides were analysed over a 150‐min gradient using Buffer A (2% acetonitrile, 0.1% formic acid), 5% Buffer B (98% acetonitrile, 0.1% formic acid). Buffer B was increased from 2 to 10% over 2 min, to 40% over 110 min, then to 85% over 1 min, maintained at 85% for 10 min and equilibrated for 14 min with 2% buffer B. Peptides were eluted at a flow rate of 300 nl min^−1^. An MS survey scan was obtained for the m/z range 300–1600. MS/MS spectra were acquired using a top 15 method, where the top 15 ions in the MS spectra were subjected to high‐energy collisional dissociation. An isolation mass window of 2.0 m/z was used for the precursor ion selection, and normalized collision energy of 27% was used for fragmentation. A duration of 5 sec was used for the dynamic exclusion. An automatic gain control target of 1 000 000 for MS and 50 000 for MS/MS was used, while maximum IT for MS was 30 msec and MS/MS was 50 msec. The system employed a resolution of 70 000 for MS and 17 500 for MS/MS. Tandem mass spectra were extracted, charge state was deconvoluted, and raw data files were converted to MGF picklists by Proteome Discoverer version 1.4 (Thermo Fisher Scientific). Data are available at PRIDE (Project https://doi.org/10.6019/pxd005408). For datasets 22–24 (Figure S1), protoplasts were generated homogenized as in Parsons *et al*. (2012), clarified by centrifugation at 3000 ***g*** for 10 min, then processed as described in Christoforou *et al*. (2016), with the exception that in the second centrifugation step at 100 000 ***g***, the supernatant was underlaid with a 25% iodixanol cushion. Spectral data are available in Table S6.

### Shotgun proteomic analysis

The MGF peaklists were each interrogated with the Mascot search engine version 2.3.02 (Matrix Science). For TripleTOF^®^ 5600 System data, a peptide tolerance of ± 50 ppm and MS/MS tolerance of ± 0.100 Da and the instrument type was set to ESI‐QUAD‐TOF. For data produced on the Q‐Exactive+, a peptide tolerance of ± 10 ppm and MS/MS tolerance of ± 0.050 Da with the instrument type set to ESI‐FTICR. Shared search parameters included variable modification of oxidation (M); fixed modifications of carbamidomethyl (C); up to one missed cleavage for trypsin. All searches were performed against Arabidopsis proteins (TAIR10) and the common Repository of Adventitious Proteins (cRAP version 1.0, The Global Proteome Machine) comprising 35 393 proteins. Mascot search results were imported into Scaffold (v4.3.4, Proteome Software) with the following filters: peptide identifications greater than 95.0% probability by the Peptide Prophet algorithm (Keller *et al*., 2002) with Scaffold delta‐mass correction; protein identifications >99.0% probability; and protein identification containing at least one identified peptide. Scaffold was used to determine average SpC for each protein in a discrete experiment (4‐week rosettes, CSCs, high‐light and low‐light conditions) by loading each replicate as a BioSample with LFDR scoring (all instruments) and protein cluster analysis parameters selected. Proteins with fewer than three spectra were discarded from analyses.

### SRM marker selection

While SRM has been adopted by the plant science community (Fan *et al*., 2012; Taylor *et al*., 2014; Duncan *et al*., 2017), relatively few resources facilitating transition selection for organelle markers exist, so extensive screening for successful transitions was required. In light of this, a minimum requirement of two marker proteins per compartment was set. Only proteins frequently experimentally localized to a compartment (Tanz *et al*., 2013) were short‐listed as SRM markers. Tissue‐specific expression was minimized by selecting proteins from genes that did not show a specific developmental profile for any of the tissues or growth stages included in this study (Winter *et al*., 2007). A total of 291 peptides from 85 proteins were assessed using total protein extracts from various Arabidopsis material. A final collection of 61 peptides from 37 proteins and 10 compartments was established (Table S3). The SRM transitions are available at PeptideAtlas (http://www.peptideatlas.org/PASS/PASS00906).

### SRM

An Agilent 1260 LC system operating in normal flow mode at 400 μl min^−1^ was coupled to an Agilent 6460QQQ Mass Spectrometer equipped with an Agilent Jet Stream source and running MassHunter version B.05.00; 10 μg of peptides was separated on an Ascentis Express Peptide C18 column [2.7 μm particle size, 160 Å pore size, 5 cm length × 2.1 mm i.d., coupled to a 5 mm × 2.1 mm i.d. guard column with similar particle and pore size, operating (CG1) at 60°C; Sigma‐Aldrich]. Peptides were ionized by using an Agilent Jet Stream source operating in positive‐ion mode with the following parameter settings: sheath gas flow = 11 l min^−1^; sheath gas temperature = 400°C; nozzle voltage = 1000 V; nebulizing pressure = 45 psi; chamber voltage = 5000 V. A 25‐min method with the following gradient was used: 95% Buffer A (2% acetonitrile, 0.1% formic acid), 5% Buffer B (98% acetonitrile, 0.1% formic acid). Buffer B is increased to 40% over 17 min, followed by an increase to 80% B in 30 sec, where it is held for 1 min. Buffer B is ramped back down to 5% in 30 sec and equilibrated for 6 min prior to the next injection. Peptide quantification was achieved by summing the integrated peak areas of two validated SRMs. Summed peaks were averaged for all peptides associated with subcellular compartments.

### SRM peptide verification

Retention times of the 61 peptides were checked, and S/N for the 122 transitions were assessed by synthesizing isotopically labelled SpikeTides comprising C‐terminal heavy Lys (^13^C_6_^15^N_2_‐Lys) or Arg (^13^C_6_^15^N_4_‐Arg; JPT Peptide Technologies GmbH). JPT also commercialized a pool of the isotope‐labelled peptides to support the rapid setup of targeted assays. A total of 0.1 pmol of each SpikeTide_L was added to 10 μg of an Arabidopsis digested total protein extract and analysed using a 25‐min gradient at 500 μl min^−1^ with a 6600 TripleTOF (SCIEX) and a nanoACQUITY UltraPerformance LC system (Waters), incorporating a C18 reverse phase column (SCIEX, 0.3 × 150 mm, 3 μm particle size). Buffer composition and gradient formation was as for SRM assays above.

### NPAS

A measure of protein abundance was developed based on data obtained from PaxDb data with augmentation from other sources that are likely enriched with low‐abundance proteins. The data imported from PaxDb were in the form of PAS normalized according to the method described by Schrimpf *et al*. (2009), which is in turn an adaptation of an earlier method (Lu *et al*., 2007). Here, the abundance of each protein is estimated by comparing the sum of SpC for its component peptides, within the generally observable size range (7–40 amino acids), with the theoretical number of peptides (i.e. a synthetic tryptic digest) in the same range, after accounting for the probabilities of peptides being observed given their lengths. Accordingly, the abundance, *a*, of a protein is estimated by taking the sum, over the *N* observed peptides, of the SpC multiplied by the length for each observed peptide *p*_*i*_ (in essence, the total of residue observations) and dividing by the sum of the lengths for the *M* theoretical, synthetic peptides *s*_*i*_ after scaling by a correction factor appropriate to each length (in essence, the expected number of observable residues):

$$a=\frac{\sum_{i}^{N}\text{count}\left(p_{i}\right).\text{length}\left(p_{i}\right)}{\sum_{j}^{M}\text{length}\left(s_{j}\right).\text{corr(length}\left(s_{j}\right))}$$

The same abundance calculation was also applied to the additional, augmenting datasets (not in PaxDb) where peptide level counts were available. Here, only canonical protein isoforms (with representative genome models from TAIR10) were considered. The peptide length correction factors (Figure S6; Table S6) were established by studying the largest dataset, number 23 (Figure S1; Table S1) and comparing the observed proportion of peptides at each length with the theoretical proportion of a synthetic TAIR10 digest. This comparison is illustrated in Figure S6, which also shows that the generally observable range of peptide lengths is about 7–42 amino acids, which corresponds to a threshold proportion of 1/3000. When peptide‐specific counts were not available, total SpC for the protein were simply scaled by the protein length.

After observing that the distribution of abundance estimates is roughly symmetric in log_10_‐space (Figure 1a and c), including for individual proteins, and that the variance in log_10_‐space across the abundance range is somewhat invariant (Figure S7), it was clearly inappropriate to take the simple arithmetic mean of abundance values. Hence, average PAS, $\hat{a}$, were calculated from the abundance values from each study by using the exponentiated mean of log_10_ *a*, from *D* separate datasets, i.e.

$$\hat{a}=10\frac{\sum_{i}^{D}\log_{10}a_{i}}{D}$$

Before the abundance scores from the additional datasets were combined with PaxDb scores, they were first aligned with the PaxDb average (as calculated above). This was achieved by selecting the proteins that were common to both PaxDb and the additional dataset, and then centring and scaling the additional dataset so that the median and standard deviation (in log_10_‐space) of the common proteins matched the PaxDB data. In this manner, the additional datasets can introduce additional proteins, but in general they are fitted to the average abundance range, even though they might represent sub‐proteome enrichments where the actual abundances of the observed proteins were inflated.

To create a final PAS, the PaxDB abundance values and those from the additional datasets were combined by using a weighted average, again in log_10_‐space. Weights were introduced, albeit in an *ad hoc* manner, to define a data priority such that additional values from the generally smaller, enriched/depleted datasets do not have an undue influence on the final average where more unbiased data are available at PaxDB. At the same time, however, when a protein is not present in PaxDB, the average will solely derive from the additional datasets. Accordingly, we set the weight to 10 for PaxDB abundances, to 2 for abundances derived from peptide level SpC, and to 1 for abundances derived from only protein total counts. A protein's PAS value is calculated over *D* available data sources as follows, where the weights *w*_*i*_ of each abundance estimate, *a*_*i*_, are 10, 2 or 1 according to the source of the estimate:

$$\text{PAS}=10\frac{\sum_{i}^{D}w_{i}\log_{10}a_{i}}{\sum_{i}^{D}w_{i}}$$

Errors in PAS values were calculated as upper and lower scores of PAS ±$10^{\hat{\sigma }}$, where $\hat{\sigma }$ is the estimated standard deviation in log_10_‐scores, using the same source weighting as above. Finally, the PAS values of all *P* observed proteins were normalized so the summation of scores is 1.0, thus creating the NPAS for each protein, *p*:

$${\text{NPAS}}_{p}=\frac{{\text{PAS}}_{p}}{\sum_{i}^{p}{\text{PAS}}_{i}}$$

### Estimating compartmental proteome abundance and enrichment in user‐submitted samples

The probability of a protein being detected during a shotgun experiment is assumed to be proportional to its cellular abundance. This means that the NPAS sum for a compartment *C* containing *N*_*C*_ proteins describes the expected proportion of total observations from that compartment (*p*_*c*_):

$$p_{c}=\sum_{i=1}^{N_{c}}{\text{NPAS}}_{i}$$

For the Arabidopsis standard proteome (TAIR10), the distribution of proteins between organelles was estimated by comparison with the HCM marker lists (i.e. the single location assignments representing 45% of each organelle proteome). NPAS values were summed for each compartment. Proteins not localized using the approach were categorized as ‘unassigned’. NPAS values were likewise summed for the ‘unassigned’ compartment. The sum of NPAS values was calculated and the compartment proportions were given as a fraction of this total. As *p*_*c*_ describes the standard organism value for a cellular compartment, and the fractions of total proteins from the compartment *C* in a user‐submitted dataset can be calculated by MMAP, then the enrichment of *C* can be modelled using a single abundance‐scaling factor, *e*_*c*_. The number of proteins from compartment *C*, identified in the new dataset by the HCM lists, as a proportion of all proteins identified in the new dataset, can be described as *q*_*c*_. Naturally, any abundance‐scaling value above 1.0 will increase the chances that a protein from compartment *C* is observed, but because the non‐compartmental proteins are in effect diluted by an enrichment we must take account of the fact that the sum of NPAS values in an enriched sample is no longer 1.0. Accordingly, we can represent the proportion of the total NPAS values that would be observed in a compartment enrichment experiment as

$$q_{c}=\frac{e_{c}p_{c}}{1+p_{c}\left(e_{c}-1\right)}$$

Solving the above for *e*_*c*_ gives the relationship between the standard compartment abundance *p*_*c*_ and the observed number of proteins *q*_*c*_:

$$e_{c}=\frac{q_{c}\left(1-p_{c}\right)}{p_{c}\left(1-q_{c}\right)}$$

The abundance‐scaling factor is converted to a new NPAS_Org, by multiplying the standard Arabidopsis compartment NPAS values by *e*_*c*_, then re‐scaling values to 1.0. This yields a new estimated relative abundance of individual proteins in a user‐submitted sample.

### MMAP integration into the SUBA web interface

The MMAP utility has been integrated as a SUBA ‘ToolBox’ and can be accessed via the SUBA4 web interface (http://suba.live). SUBA4 utilizes the database programming language SQL (Structured Query Language). The NPAS and HC‐marker lists have been integrated into the SUBA server. The graphical use interface was written in Dynamic Hyper Text Markup Language that makes use of Asynchronous JavaScript and JSON (AJAX) to interact with the SUBA server. Upon submission of data (a list of AGIs), the script assesses the provided identifiers for matches within the HC‐marker list.

## Supporting information


**Figure S1.** Newly acquired and previously published peptide spectral data used to generate NPAS.
**Figure S2.** Previously published protein spectral data used to generate NPAS.
**Figure S3.** Correlation between ESTs and NPAS.
**Figure S4.** Estimation of subcellular composition using NPAS, compared with estimates from SpC and SRM, for individual examples of plant material grown under low‐light conditions.
**Figure S5.** Comparison of summed SpC for subcellular locations in different plant material compared with abundance‐scaling factors.
**Figure S6.** Calculation of peptide length‐correction factors.
**Figure S7.** Relationship between log_10_‐scale mean and dispersion of NSAF values from PaxDb.

**Table S1.** Publications containing supplemental SpC data used in NPAS.
**Table S2.** Normalized PAS.
**Table S3.** HC‐marker collections used to assign proteins to subcellular locations.
**Table S4.** Overview of SRM transitions.
**Table S5.** SRM signal intensity values for all target peptides after normalization to stable isotope‐labelled standards.
**Table S6.** Peptide spectral matches for datasets 22–24 in Figure S1.
