# Supplementary material for: Multiple marker abundance profiling: combining selected reaction monitoring and data‐dependent acquisition for rapid estimation of organelle abundance in subcellular samples
Source: Plant J. 2017 Nov 20;92(6):1202–17. doi: 10.1111/tpj.13743 (PMC5863471; doi:10.1111/tpj.13743)
Supplement: Supplementary file 1 — Figure S1. Newly acquired and previously published peptide spectral data used to generate NPAS. Figure S2. Previously published protein spectral data used to generate NPAS. Figure S3. Correlation between ESTs and NPAS. Figure S4. Estimation of subcellular composition using NPAS, compared with estimates from SpC and SRM, for individual examples of plant material grown under low‐light conditions. Figure S5. Comparison of summed SpC for subcellular locations in different plant material compared with abundance‐scaling factors. Figure S6. Calculation of peptide length‐correction factors. Figure S7. Relationship between log10‐scale mean and dispersion of NSAF values from PaxDb. [file TPJ-92-1202-s001.pdf]

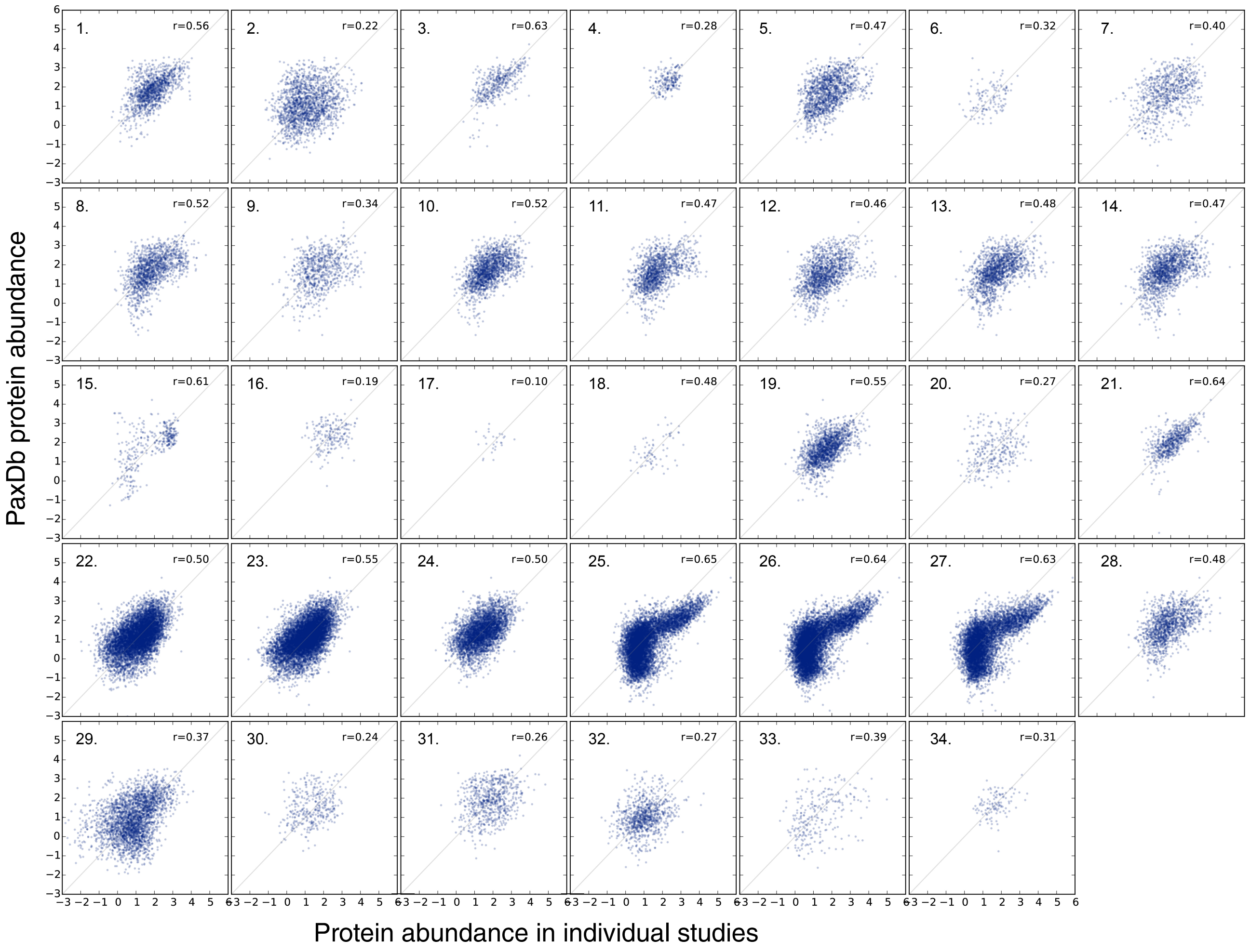

Figure S1. Newly acquired and previously published petide spectral data used to generate NPAS. Spectral data was centered and scaled to exitingtissue proteome data housed at paxdb.org. The vertical axes show the log10-average of PaxDb NSAF values and the horizontal axes show the abundance estimates for each additional dataset calculated using peptide-specific spectral counts. Numbers refer to PubMed Unique Identifier (PMID), detailed in Table S1.

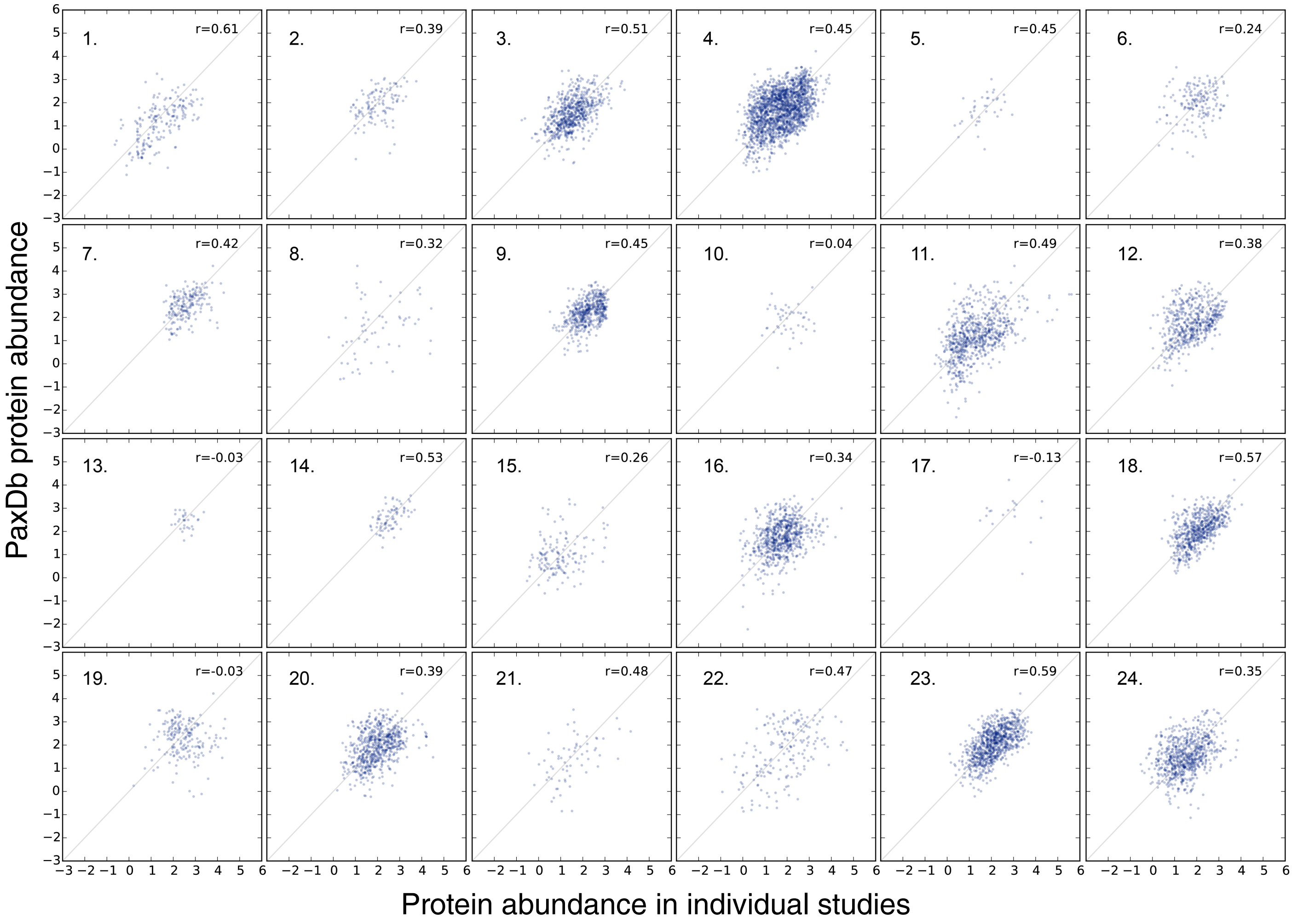

Figure S2. Previously published protein spectral data used to generate NPAS. Spectral data was centered and scaled to existing tissue proteome data housed at paxdb.org. The vertical axes show the log10-average of PaxDb NSAF values and the horizontal axes show the abundance estimates for each additional dataset calculated using whole-protein spectral counts. Numbers refer to PMID, detailed in Table S1.

Figure S3.

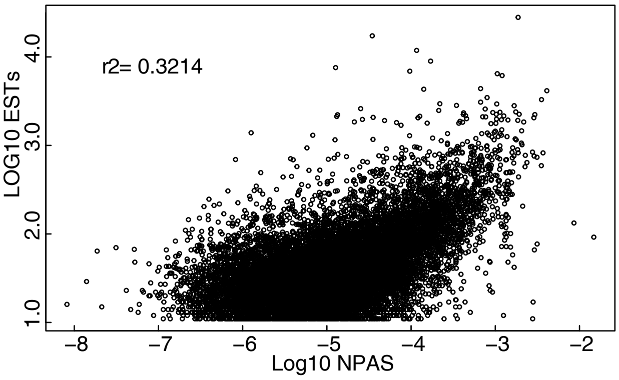

Figure S3. Correlation between ESTs and NPAS.
Counts of expressed sequence tags (ESTs) from TAIR10 were plotted against NPAS, after removal of genes represented by 1 or fewer ESTs. This showed limited correlation between transcript and protein abundance.

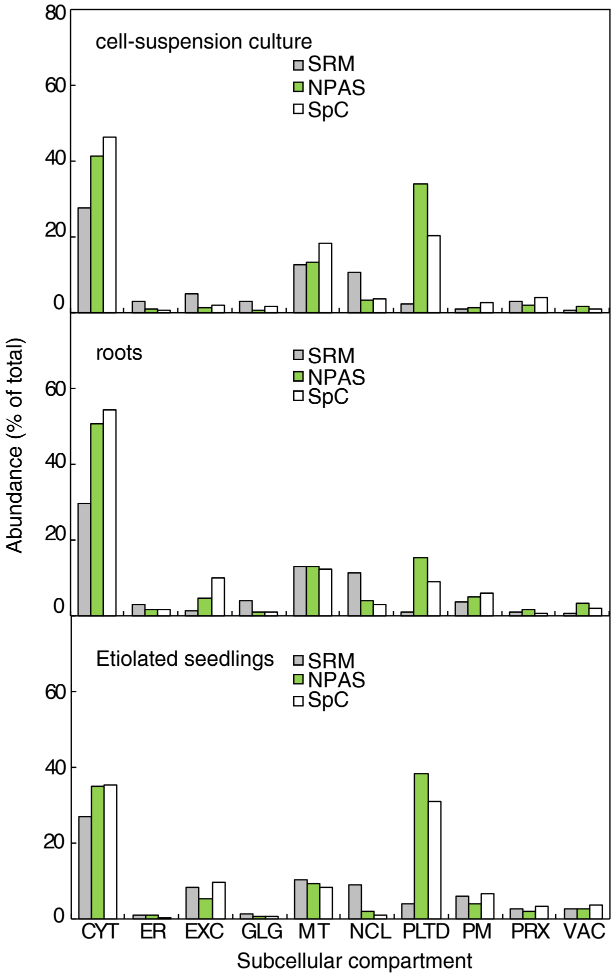

Figure S4. Estimation of subcellular composition using NPAS, compared to estimates from SpC and SRM, for individual examples of plant material grown under low-light conditions. Analysis was performed as for Fig. 4, in which mean results for the low-light growth condition group are shown.

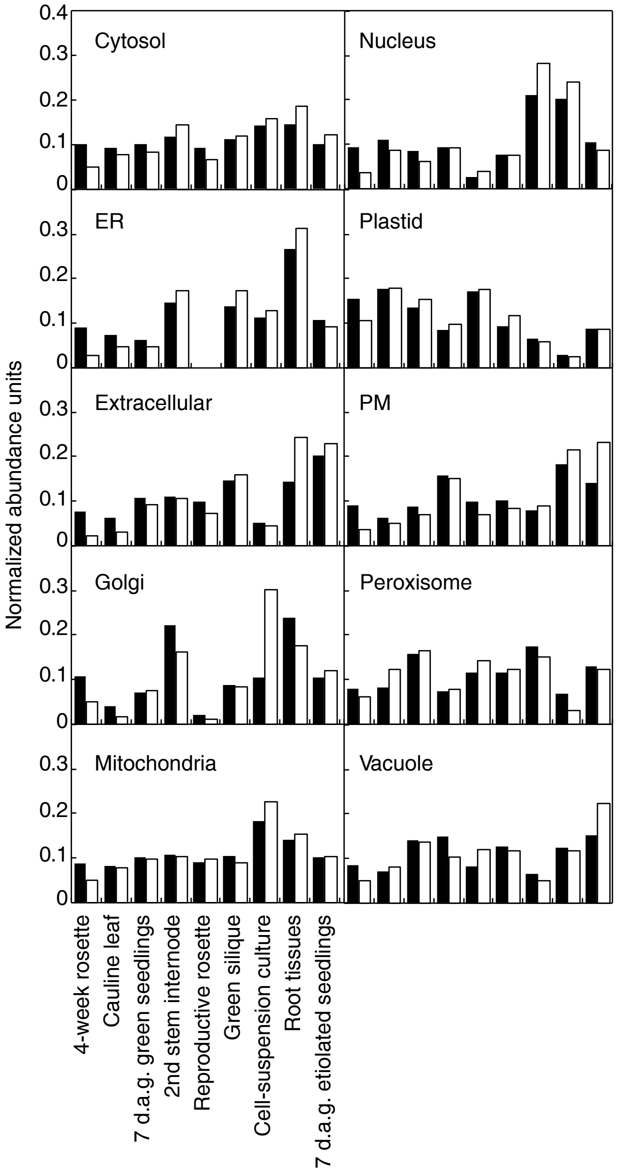

Figure S5. Comparison of summed SpC for subcellular locations in different plant material compared to abundance-scaling factors. Changes in summed SpC (white) were compared to changes in abun dance-scaling (black) factor in different plant material for all subcellular locations. The abundance-scaling factor is a unique value for each subcellular location in every dataset. It describes the amount of compart ment enrichment or depletion required to explain the number of compartment proteins detected in a user's dataset, given the expected ratios of compartment proteins in the Arabidopsis proteome. Summed SpC for each compartment reflect actual changes in compartment abundance, so changes in abun dance-scaling factors should be comparable to changes in summed SpC.

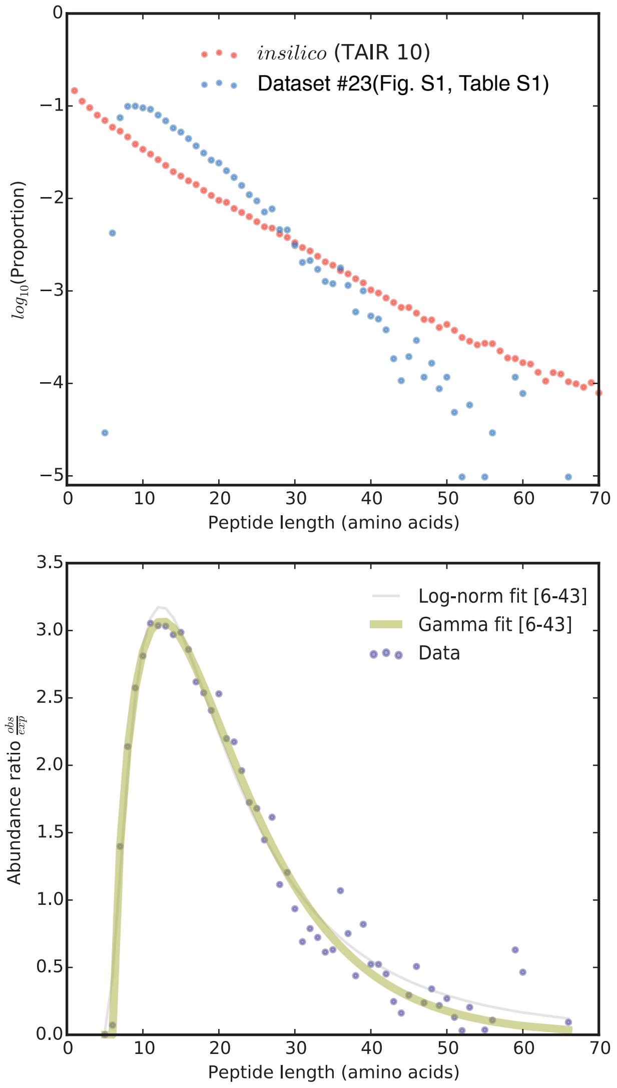

Figure S6. Calculation of peptide length-correction factors.
The proportional abundance of peptides with different numbers of amino acids was computed for both dataset #23 and for a theoretical (in silico) tryptic digest of the TAIR 10 Arabidopsis proteome. A scatter plot of these proportions (top) shows how the experimental peptide detectability, at each length, differs from the ideal/theoretical proportion. The range of lengths for which peptides were deemed to be generally detectable is 7-42. The lower limit is at a sharp drop-off in detectability and above this range the experimental proportion is both low and somewhat noisy. Calculation of the ratio between these two proportions at each length shows a distribution which fits a gamma function (bottom). The parameters of the gamma function were optimized using scipy.optimise.curve_fit from the SciPy Python library (www.scipy.org) and considered lengths in the range 6-42. Here the near-zero value for length 6 was useful to restrain the curve fitting, even if the abundance at this length is too low for practical use. The fitted ratios from the gamma function (yellow line) were then used as correction factors for the corresponding peptide lengths.

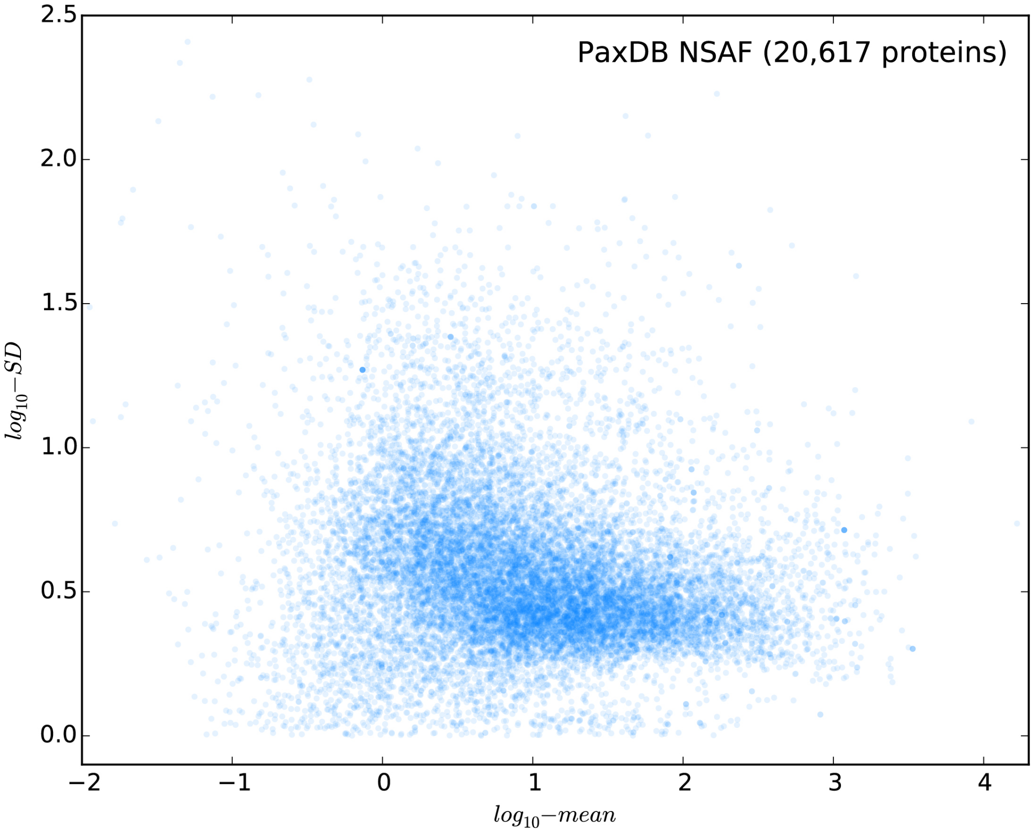

Figure S7. Relationship between log10-scale mean and dispersion of NSAF values from PaxDb. The NSAF values from PaxDb provide multiple estimates for protein abundance from various studies. An analysis of the mean and standard deviation (SD) of NSAF values, i.e. for each protein across the different studies, was performed to show any general relationship between these two statistics. While the standard deviation generally increases as the mean increases, calculating the mean and standard deviation of the log10(NSAF) for each protein (blue dots) shows that the dispersion is somewhat invariant when using a log10 scale. There is a slight trend for a higher log10-SD at lower abundance values, as might be expected from decreased precision of low-abundance peptide counts. Nonetheless, using the log10(NSAF) removes the first-order dependence between score mean and standard deviation.
