# Supplementary material for: Multiple marker abundance profiling: combining selected reaction monitoring and data‐dependent acquisition for rapid estimation of organelle abundance in subcellular samples
Source: Plant J. 2017 Nov 20;92(6):1202–17. doi: 10.1111/tpj.13743 (PMC5863471; doi:10.1111/tpj.13743)
Supplement: Supplementary file 3 [file TPJ-92-1202-s003.docx]

**Supporting Information Legends**

**Figure S1. Newly acquired and previously published peptide spectral data used to generate NPAS.** Spectral data was centered and scaled to existing tissue proteome data housed at paxdb.org. The vertical axes show the log10-average of PaxDb NSAF values and the horizontal axes show the abundance estimates for each additional dataset calculated using peptide-specific spectral counts. Numbers refer to PubMed Unique Identifier (PMID), detailed in Table S1.

**Figure S2. Previously published protein spectral data used to generate NPAS.** Spectral data was centered and scaled to existing tissue proteome data housed at paxdb.org. The vertical axes show the log10-average of PaxDb NSAF values and the horizontal axes show the abundance estimates for each additional dataset calculated using whole-protein spectral counts. Numbers refer to PMID, detailed in Table S1.

**Figure S3. Correlation between ESTs and NPAS.**

Counts of expressed sequence tags (ESTs) from TAIR10 were plotted against NPAS, after removal of genes represented by 1 or fewer ESTs. This showed limited correlation between transcript and protein abundance.

**Figure S4.** **Estimation of subcellular composition using NPAS, compared to estimates from SpC and SRM, for individual examples of plant material grown under low-light conditions.** Analysis was performed as for Fig. 4, in which mean results for the low-light growth condition group are shown.

**Figure S5. Comparison of summed SpC for subcellular locations in different plant material compared to abundance-scaling factors.** Changes in summed SpC (white) were compared to changes in abundance-scaling (black) factor in different plant material for all subcellular locations. The abundance-scaling factor is a unique value for each subcellular location in every dataset. It describes the amount of compartment enrichment or depletion required to explain the number of compartment proteins detected in a user’s dataset, given the expected ratios of compartment proteins in the Arabidopsis proteome. Summed SpC for each compartment reflect actual changes in compartment abundance, so changes in abundance-scaling factors should be comparable to changes in summed SpC.

**Figure S6. Calculation of peptide length-correction factors.**

The proportional abundance of peptides with different numbers of amino acids was computed for both dataset #23 and for a theoretical (in silico) tryptic digest of the TAIR 10 Arabidopsis proteome. A scatter plot of these proportions (top) shows how the experimental peptide detectability, at each length, differs from the ideal/theoretical proportion. The range of lengths for which peptides were deemed to be generally detectable is 7-42. The lower limit is at a sharp drop-off in detectability and above this range the experimental proportion is both low and somewhat noisy. Calculation of the ratio between these two proportions at each length shows a distribution which fits a gamma function (bottom). The parameters of the gamma function were optimized using scipy.optimise.curve_fit from the SciPy Python library (www.scipy.org) and considered lengths in the range 6-42. Here the near-zero value for length 6 was useful to restrain the curve fitting, even if the abundance at this length is too low for practical use. The fitted ratios from the gamma function (yellow line) were then used as correction factors for the corresponding peptide lengths.

**Figure S7. Relationship between log_10_-scale mean and dispersion of NSAF values from PaxDb.** The NSAF values from PaxDb provide multiple estimates for protein abundance from various studies. An analysis of the mean and standard deviation (SD) of NSAF values, i.e. for each protein across the different studies, was performed to show any general relationship between these two statistics. While the standard deviation generally increases as the mean increases, calculating the mean and standard deviation of the log_10_(NSAF) for each protein (blue dots) shows that the dispersion is somewhat invariant when using a log_10_ scale. There is a slight trend for a higher log_10_-SD at lower abundance values, as might be expected from decreased precision of low-abundance peptide counts. Nonetheless, using the log_10_(NSAF) removes the first-order dependence between score mean and standard deviation.

**Table S1. Publications containing supplemental spectral count data used in NPAS.** Table S1 lists sources for all data used to generate NPAS. For some publications, peptide spectral match data was available, for other only the total spectral count per protein was given (“spectral match level”). Spectral data for each publication has been scaled to Arabidopsis data from paxdb.org, and represented graphically in Fig. S1 (“Data number Fig. S1”).

**Table S2. Normalized Protein Abundance Scores.** Newly-acquired data (Project DOI: 10.6019/PXD007246, 10.6019/PXD007245, 10.6019/PXD007247) and published data (Table S1) was median-centered and scaled to existing tissue proteome data in paxdb.org, as detailed in the experimental procedures, and standard deviation calculated for all proteins represented in more than one study. Data was taken from representative gene models only. Normalization to the PAS total gave NPAS. Standard deviation (log10) was added or subtracted to PAS (log10), then normalized to the PAS total to give minimum and maximum error boundaries for NPAS. Final values used in MMAP are displayed in bold.

**Table S3.** **High-confidence (HC) marker collections used to assign proteins to subcelular locations.** Markers comprise the 45% most confidently-localized proteins to each of the major subcellular locations, as previously defined by Hooper *et al*., 2014, after manual editing of data-rich proteins assigned multiple times to multiple subcellular locations.

**Table S4. Overview of SRM transitions.** Further details are available at SRM transitions are available at PeptideAtlas (http://www.peptideatlas.org/PASS/PASS00906)

**Table S5. SRM signal intensity values for all target peptides after normalization to stable isotope-labelled standards.** Further details are available at SRM transitions are available at PeptideAtlas (<http://www.peptideatlas.org/PASS/PASS00906>).

**Table S6. Peptide spectral matches for datasets 22 – 24 in Figure S1.**
